# Leveraging the NIH Toolbox for Early Detection of Cognitive Impairment in Primary Care: The Toolbox Detect Trial

**DOI:** 10.1002/alz70858_101295

**Published:** 2025-12-24

**Authors:** Stephanie Ruth Young

**Affiliations:** ^1^ Northwestern University Feinberg School of Medicine, Chicago, IL, USA

## Abstract

**Background:**

Primary care visits offer a critical opportunity for early detection of cognitive impairment (CI) in adults over age 65. Yet, less than half of patients with CI are identified in these settings. To address barriers to screening and improve detection rates, the NIH‐funded Toolbox Detect initiative launched MyCog, a self‐administered, tablet‐based cognitive screener that integrates with the electronic health record (EHR) and provides turnkey decision‐making support. MyCog leverages well‐validated self‐administered neuropsychological measures. The aim of the project is to evaluate MyCog's effectiveness in improving the timely detection and management of cognitive decline in primary care, alongside its feasibility, implementation fidelity, and associated costs.

**Method:**

Enrollment includes Northwestern Medicine Primary Care clinics, defined as clinics with at least one clinician performing Medicare Annual Wellness Visits (AWVs) who agree to trial participation and randomization. Eligible patients are aged 65+ and complete an AWV at an enrolled clinic with a participating clinician. Clinics are randomized to either implement MyCog with EHR integration or follow enhanced usual care. The trial also extends to ACCESS Community Health Network, a Federally Qualified Health Center (FQHC) network, where the protocol is being replicated to assess its adaptability for diverse populations.

**Result:**

To date, Toolbox Detect has enrolled 31 clinics serving 28,433 patients total with over 6,796 patients in the MyCog intervention arm. Clinic feedback has informed improvements to the app's usability, including redesigns to enhance user experience and workflow integration while preserving its psychometric properties and fidelity to study procedures. Early implementation findings highlight the feasibility and acceptability of the MyCog approach, with insights into its integration into primary care workflows and its adaptation for community health centers serving diverse patient populations.

**Conclusion:**

MyCog represents a scalable, evidence‐based cognitive screening tool tailored to primary care workflows. By addressing common barriers to CI screening—including time constraints, workflow disruptions, and manual data entry—the project aims to improve the early detection, diagnosis, and referral for CI and dementia. Findings from this trial will inform best practices for introducing novel technologies into primary care and community health settings, with attention to provider, patient, and care partner unmet needs.

